# Supramolecular Materials in Extreme Environments: Balancing Stability and Dynamics

**DOI:** 10.3390/polym18121458

**Published:** 2026-06-11

**Authors:** Yiwa Wang, Chao Yu, Jingnan Li, Jianfeng Cheng, Xiuming Liu, Songbao Fu

**Affiliations:** China National Offshore Oil Corporation (CNOOC), Institute of Chemicals & Advanced Materials, Beijing 102200, China; wangyw46@cnooc.com.cn (Y.W.); yuchao12@cnooc.com.cn (C.Y.); lijn16@cnooc.com.cn (J.L.); chengjf4@cnooc.com.cn (J.C.); liuxm44@cnooc.com.cn (X.L.)

**Keywords:** supramolecular materials, stability–dynamics balance, extreme environments, non-covalent interactions

## Abstract

The development of supramolecular materials has opened up unprecedented opportunities for smart, responsive systems. Yet, their practical application in extreme environments—deep space, deep sea, polar regions, high-temperature and high-pressure reservoirs—is fundamentally challenged by the inherent trade-off between structural stability and dynamic adaptability. This review addresses this core issue by presenting a comprehensive framework for understanding and overcoming the stability–dynamism mismatch under harsh condition. We systematically analyze the molecular mechanisms by which severe factors disrupt non-covalent networks. Based on these insights, we outline four universal molecular design strategies that re-establish the balance, and summarize engineering applications across aerospace, marine, energy, and polar exploration. Beyond offering a comprehensive roadmap for rational material design, this review highlights persistent challenges—including multi-field coupling failure mechanisms, industrialization barriers, and the limitations of current systems—and outlines future directions. By bridging fundamental chemistry with extreme environment engineering, this work aims to guide the next generation of supramolecular materials that can reliably serve in the most demanding operational scenarios.

## 1. Introduction

### 1.1. Supramolecular Materials

Supramolecular materials, sometimes referred to as non-covalent responsive materials, are functional systems constructed using non-covalent interactions between molecules, providing access to stimuli-sensitive behaviors and widely customizable mechanical properties. The key features of supramolecular materials, in contrast to conventional polymeric materials based on strong covalent connections, are their structures’ reversibility, dynamism, and environmental adaptability [1,2]. Although the bond energies of these non-covalent interactions are generally much lower than those of covalent bonds, their synergistic combination allows for the creation of advanced materials with complex functions and excellent mechanical properties [3].

Pedersen’s groundbreaking research on crown ethers is the historical source of the idea of supramolecular chemistry. The fundamental concepts of “molecular recognition” and “self-assembly” were later developed by Cram, Lehn, and others. Lehn defined supramolecular chemistry as “chemistry beyond the molecule,” concentrating on the study of ordered, functionalized chemical systems created by non-covalent interactions [1,2]. In the 21st century, the research focus has shifted from “understanding self-assembly” to “functional applications”, giving rise to supramolecular polymer materials as a multidisciplinary field [4].

Supramolecular materials offer several distinct advantages. First, their reversibility and self-healing capabilities allow microscopic damage to be repaired through dynamic bond breaking and reformation, greatly extending service life [5,6]. Second, they respond sensitively to environmental changes such as temperature, pH, light, electric fields, and ionic strength [7,8]. Third, the dynamic nature of non-covalent networks provides processability and recyclability comparable to thermoplastics, enabling green recycling and circular use [1]. Fourth, the capacity to precisely build molecular structures to control macroscopic features including mechanical, optical, and electrical properties at the nanoscale is made possible by functional programmability [4].

Non-covalent interactions come in diverse forms, offering a multitude of possibilities for supramolecular material design. Hydrogen bonding was one of the first non-covalent interactions employed with supramolecular polymers. Lehn et al. originally reported linear supramolecular polymers based on triple hydrogen bonds in 1990 [1]. The ureidopyrimidinone (UPy) quadruple hydrogen-bonding unit, which has a dimerization constant as high as 6 × 10^7^ M^−1^ in CDCl_3_, was later developed by Meijer et al. and became a common building block for supramolecular polymers [1,4]. Metal coordination bonds represent another important class of non-covalent interactions. Their bond energies can be tuned within the range of 10–400 kJ·mol^−1^, offering the dual advantages of high strength and reversibility [3]. Host–guest interactions, on the other hand, are non-covalent bonding mechanisms based on the principle of molecular recognition, with cyclodextrins, crown ethers, cucurbiturils, calixarenes, and pillararenes being commonly used host molecules [1,8]. π–π stacking interactions, commonly found in conjugated aromatic systems, can promote columnar stacking and fiber network formation when peripheral directing groups are introduced [1]. Electrostatic and hydrophobic interactions are also important driving forces in the construction of supramolecular materials, playing a key role particularly in bio-related systems and self-healing hydrogels [6,9].

Currently, the research landscape of supramolecular materials has expanded from the initial focus on host–guest chemistry and molecular recognition to numerous cutting-edge fields such as self-healing elastomers, biomimetic adhesives, flexible electronics, organic optoelectronic devices, energy storage and conversion, and drug delivery [4,8,10]. In the field of molecular electronics, researchers have utilized π–π-stacking dimers to achieve sub-nanometer precision in quantum interference control, providing new insights for the development of adaptive nanocircuits [4]. In the biomedical field, supramolecular polymers based on hydrogen bonding and host–guest interactions can enable the dynamic, responsive release of drugs in complex biological environments, significantly improving therapeutic efficacy [8]. In the field of sustainable materials, elastomers designed through the synergistic combination of metal coordination and hydrogen bonding simultaneously achieve high toughness, self-healing, and recyclability [5,6]. Notably, the physical networks formed by non-rubber components in natural rubber (such as proteins and phospholipids) through non-covalent interactions have a significant impact on the mechanical properties and storage hardening of rubber, providing a natural template for the design of bio-based supramolecular materials [9].

### 1.2. Strategic Demand for Supramolecular Materials in Extreme Environments

As national strategies extend into deeper, more distant, and more extreme environments on Earth, unprecedented challenges and demands have arisen for smart materials capable of operating under such harsh conditions. In the field of deep-space exploration, spacecraft components must withstand extreme temperature fluctuations ranging from −180 °C to 150 °C, as well as high vacuum and cosmic radiation [11]. In deep sea fengineering, materials must withstand hydrostatic pressures of up to approximately 110 MPa at the bottom of the Mariana Trench, a high salinity of approximately 3.5 wt%, and low temperatures of 2 °C, i.e., near freezing [12,13]. In high-end petrochemical and energy extraction applications, materials must operate stably over extended periods in environments characterized by temperatures exceeding 200 °C, pressures of 30 MPa, and corrosive conditions involving strong acids and alkalis [11,14]. These extreme scenarios demand not only passive structural stability and durability but also active intelligent capabilities—sensing, sealing, or adaptive structural responses to external stresses—while maintaining stable dynamic responsiveness. Meeting these dual requirements constitutes the core material bottleneck for advanced equipment and major engineering projects.

### 1.3. The “Stability–Dynamism” Paradox for Supramolecular Materials in Extreme Environments

Despite significant progress in non-covalent responsive materials, the inherent stability–dynamism paradox of non-covalent bonds is drastically amplified in extreme environments (e.g., extreme cold below −80 °C, high temperatures above 200 °C, high pressure, and strong corrosion) [7,9]. At low temperatures, molecular chain motion is “frozen,” free water crystallizes, and the rate of dynamic bond exchange drops sharply, causing the material to lose its flexibility and self-healing capability. At high temperatures, thermal motion intensifies, and the dissociation rate of weak interactions such as hydrogen bonds far exceeds the recombination rate, leading to the destruction of the network structure and a decrease in modulus. In highly corrosive environments, ion shielding effects and competition from water molecules specifically disrupt non-covalent bonds, resulting in material failure. Therefore, how to re-establish the stability–dynamic equilibrium under extreme conditions, thereby enabling materials to achieve long-term stable service and efficient dynamic response under harsh conditions, has become a core scientific problem urgently requiring resolution in this field.

Unlike previous reviews that focused on specific environmental factors [9], this work establishes an integrated framework that connects multi-field coupling failure mechanisms to validated engineering applications. This review highlights the molecular causes of stability–dynamic imbalance under different extreme conditions and the resulting universal design strategies, and describes potential applications in common extreme engineering scenarios and upcoming challenges.

## 2. Fundamentals of Supramolecular Materials: Structural and Dynamic Characteristics

### 2.1. Basic Characteristics of Supramolecular Materials

A supramolecular polymeric material is defined as “an array of polymeric units linked by highly directed and reversible non-covalent interactions, exhibiting polymeric properties in both solution and the bulk” [15]. Unlike traditional covalent materials, the “degree of polymerization” of supramolecular materials is influenced by concentration, temperature, and competing solvents, exhibiting dynamic and reversible characteristics [16,17].

From a macroscopic perspective, supramolecular polymeric materials can exhibit two extreme states: one is a randomly entangled coil structure, which possesses the mechanical properties of plastics and elastomers but, due to its reversible monomer–polymer transition, offers excellent processability, recyclability, and self-healing capabilities [18]. The other is a shape-stable and highly ordered fibrous structure formed by self-assembly of designed subunits [17]. A vast design space exists between these two extreme states, offering abundant possibilities for the development of functional materials [19].

Lehn defined supramolecular chemistry as “chemistry beyond the molecule,” with its core lying in the storage and processing of information—the arrangement of functional groups within the molecular structure encodes assembly instructions, while non-covalent interactions execute these instructions, driving the system to spontaneously form aggregates with specific structures and functions [20]. This “information paradigm” provides the theoretical foundation for the rational design of supramolecular materials [21].

### 2.2. Thermodynamic Mechanisms of Supramolecular Polymerization

In contrast to conventional covalent polymerization, supramolecular polymerization follows the equilibrium thermodynamics of non-covalent bonds. There are primarily three thermodynamic mechanisms in supramolecular polymerization [22].

The isodesmic mechanism is the most fundamental type, where the equilibrium constants for each monomer addition step are equal and independent of chain length. The degree of polymerization (DP) and monomer concentration follow the relationship DP ~ (K·C)^1/2^. Typical isodesmic systems include weak hydrogen bonding, π–π stacking, and certain metal coordination systems, characterized by the absence of significant nucleation barriers and polydisperse assemblies [15].

The ring-chain equilibrium mechanism describes the dynamic equilibrium between linear supramolecular polymers and cyclic oligomers. In dilute solutions, cyclic species dominate; as concentration increases, the proportion of linear polymers rises [23]. This mechanism is crucial for understanding concentration-dependent behavior.

Cooperative mechanisms divide polymerization into nucleation and growth stages. The nucleation constant is much smaller than the growth constant, leading to a sharp increase in polymerization degree above a critical concentration, with an S-shaped kinetic curve [24]. Typical cooperative systems include UPy quadruple hydrogen-bond arrays, π–π stacking of perylene bisimides, and certain metal–ligand systems [25,26]. The cooperative mechanism provides a theoretical foundation for “living supramolecular polymerization” and “seed-growth” strategies [27].

Under extreme conditions, factors such as temperature, solvent polarity, and ionic strength can significantly alter the polymerization constant, potentially shifting the polymerization mechanism—this is the thermodynamic root cause of the stability–dynamism imbalance.

**Figure 1:**
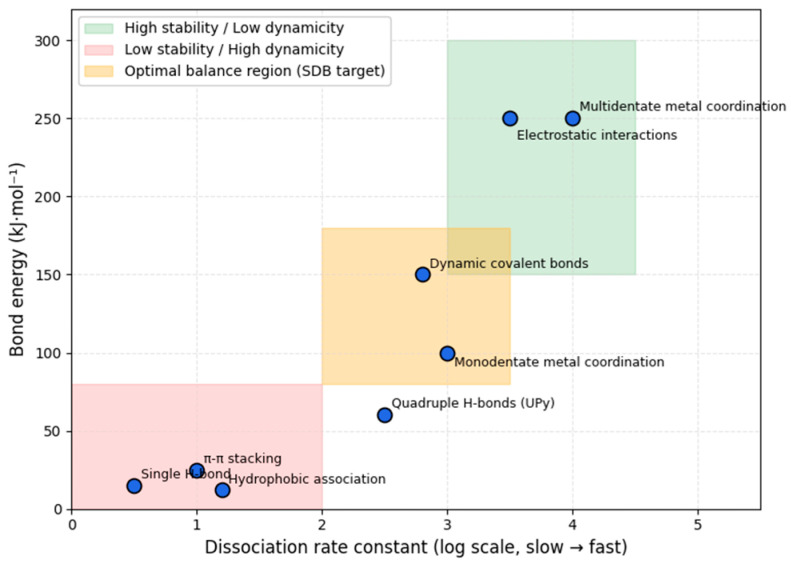
Correlation between bond energy and dissociation kinetics for typical non-covalent interactions.

Figure 1 shows the relationship between bond energy and dissociation kinetics for typical non-covalent interactions. Figure 1 illustrates the general trend that non-covalent interactions with higher bond energy exhibit slower dissociation kinetics, whereas low-energy interactions (e.g., weak hydrogen bonds) undergo rapid exchange. This trade-off underpins the orthogonal design strategy described in Section 4.

To rationally navigate this landscape, precise control over hydrogen bonding—the most versatile supramolecular handle—is required. Table 1 classifies hydrogen bonds into four categories based on binding energy, ranging from very strong (100–167 kJ/mol) to weak (<20 kJ/mol) [14,15]. This refined classification provides a quantitative framework for selecting building blocks.

While Table 1 provides a static view, the actual dissociation rate constant ($k_{off}$) is not dictated by bond energy alone. It is profoundly modulated by activation entropy, cooperativity, pre-organization, and chelate effects. For instance, multivalent interactions can reduce $k_{off}$ by orders of magnitude compared to monovalent analogs with similar free energy changes ($△G^{0}$) [28]. Recent advances exemplify this principle: Niu et al. engineered hydrogen-bond arrays with optimized geometries that achieved remarkable thermal stability (up to 120 °C) and mechanical strength (true fracture stress of 1.1 GPa). These findings demonstrate that although the thermodynamic constraint ($k_{off}\propto exp(-△G^{0}/RT)$) is universal, strategic molecular design can effectively decouple stability from rigidity, mitigating the stability–dynamism dilemma.

### 2.3. Multiscale Structural Hierarchy of Supramolecular Materials

The structure–property relationship of supramolecular materials can be understood across three scales [22].

Molecular scale: This involves the chemical structure of monomers, types and arrangements of non-covalent groups (e.g., DADA vs. AADD hydrogen bonding arrays), binding constants, and dissociation rate constants [29]. This level determines intrinsic properties—bond energy, directionality, selectivity, and kinetics. For example, the K_dim_ of UPy quadruple hydrogen bonds reaches 6 × 10^7^ M^−1^, whereas ADA type triple hydrogen bonds are only 10^2^–10^3^ M^−1^ [30]. This difference directly determines the mechanical strength and thermal stability of the resulting supramolecular polymers [31,32].

Network level: This involves crosslinking density, chain length distribution, network topology (linear, star, brush), and covalent backbone properties (molecular weight, T_g_, flexibility) [33]. This level determines macroscopic mechanical behavior and dynamic response characteristics. For instance, a polysiloxane backbone with a low T_g_ provides segmental motion capability to supramolecular elastomers at low temperatures [34], whereas a polystyrene backbone with a high T_g_ results in a glassy state at room temperature [35]. Selecting an appropriate backbone is key to achieving stability–dynamism balance within a specific temperature range [36]. In the work of Stach et al., a flexible PEG cross linker enabled hydrogel formation at low solid content (1 wt%), demonstrating the importance of backbone flexibility [37].

Mesoscopic level: This includes microphase separated structures (hard soft microdomains), nanofiber/micelle assemblies, crystalline domains, and hierarchical ordered structures [38]. Wang et al. confirmed through SAXS and WAXD that the polyurethane urea elastomer SPUU-DA (supramolecular polyurethane-urea elastomer with rigid DABA and flexible ADH segments), combining rigid (DABA) and flexible (ADH) supramolecular segments, exhibits a phase separation period of ~10.6 nm and undergoes strain induced crystallization during tensile loading, endowing the material with a fracture toughness of 1.2 GJ·m^−3^ and a fracture energy of 282.5 kJ·m^−2^ [18]. Typical mesoscopic structures also include core–shell nanostructures (hydrophobic core as physical crosslinking point, hydrophilic shell for solvation and dynamic response) [39], rigid–flexible microphase separation (rigid microdomains as sacrificial bonds to dissipate energy while the flexible continuous phase maintains network integrity) [18,40], and hierarchical porous structures [41].

These three levels are mutually coupled and act synergistically. For example, supramolecular elastomers with a low T_g_ backbone (molecular level) and high density hydrogen bond crosslinking (network level) can retain segmental mobility at low temperatures while maintaining network integrity [31]. However, when the ambient temperature is too low, segmental motion is frozen; even though hydrogen bonds remain intact, the material’s dynamic response is completely lost—this is the “low temperature imbalance” mechanism [42].

### 2.4. Dynamic Properties of Supramolecular Systems

The concept of “Constitutional Dynamic Chemistry” (CDC), proposed by Lehn in 2007, expanded supramolecular chemistry from static molecular recognition to dynamic, environment adaptive chemical systems [43]. Within this framework, supramolecular systems can achieve dynamic structural adjustments through reversible non-covalent bonds and respond to external stimuli via component exchange and reorganization, realizing “self adaptive” functions [20]. Lehn described the evolution of supramolecular chemistry through three overlapping phases: (i) molecular recognition and its corollaries; (ii) self assembly and self organization; and (iii) constitutional dynamics (adaptation and evolution) [43].

Dynamic Covalent Chemistry (DCvC) further extended this concept [44]. By introducing reversible covalent bonds (imine, boronate, disulfide), materials gain dynamic reversibility while retaining the mechanical strength of covalent bonds [45]. Wayment et al. highlighted that synergy between DCvC and supramolecular chemistry—including metal–ligand coordination, host–guest binding, and hydrogen bonding—enables construction of polymers with enhanced structural order and functionality [45].

Aida, Meijer, and Stupp, noted that intermediate bond lifetimes present opportunities to create materials that combine adaptability, responsiveness, self-healing, and unique processing properties [17]. Highly ordered supramolecular polymers can undergo distinct transitions between short- and long-bond-lifetime states due to their self-assembly mechanisms [19]. Yu et al. utilized host–guest interactions between β-cyclodextrin and camptothecin to construct GSH-responsive supramolecular nanodrugs; the assemblies remain stable in physiological environments but rapidly dissociate to release the drug under high GSH conditions in tumor cells [39]. The dynamic nature of supramolecular systems also enables self-healing, as demonstrated by Cordier et al., who reported a supramolecular rubber that self-heals within minutes at room temperature through reversible hydrogen bonding [46].

Table 2 provides a critical comparison of supramolecular materials with conventional polymers, covalent adaptable networks (CANs, including vitrimers), and hybrid systems. CANS rely on associative exchange mechanisms within covalent networks, combine the mechanical integrity of thermosets with the reprocessability of thermoplastics, providing a benchmark for evaluating trade-offs between dynamicity and mechanical strength in supramolecular designs [47,48,49].

### 2.5. From Fundamental Framework to Instability Mechanisms

Having established the fundamental framework, we now explore how the stability–dynamic equilibrium is disrupted in extreme environments. Stability refers to the material’s ability to maintain structural integrity of its non-covalent network and resist irreversible damage under extreme stress; dynamism refers to the ability to undergo reversible dissociation and reorganization of non-covalent bonds under stress. High bond energy yields high stability but poor dynamism, while low bond energy offers excellent dynamism but insufficient stability—this inherent contradiction is key to understanding the subsequent destabilization mechanisms.

Different extreme environments disrupt this balance through distinct mechanisms: low temperatures “freeze” segmental motion, high temperatures accelerate bond dissociation, high salinity masks electrostatic interactions, and high pressure compresses assembled structures. Seiffert and Sprakel emphasized that the dynamics of supramolecular polymer networks are governed by two characteristic timescales: the lifetime of supramolecular bonds and the relaxation time of polymer chains [22]. When these timescales become mismatched under extreme conditions, material performance degrades. Furthermore, synergistic failure effects from coupling multiple factors in real-world applications pose a severe challenge.

Having established this multiscale framework, the following section (Section 3) will examine how each type of extreme environment specifically disrupts these structural levels, leading to characteristic failure modes.

## 3. The Root of Imbalance: The Origin of the Stability–Dynamism Paradox in Extreme Environments

It should be noted that the service environments in which materials operate are typically complex systems involving the coupling of multiple factors. However, to clearly elucidate the independent failure mechanisms of each environmental factor on non-covalent bond networks, this section will first analyze the imbalance mechanisms under single extreme environments—such as low temperature, high temperature, high salinity/corrosion, and high pressure—one by one.

Building on this foundation, Section 3.6 will further explore the patterns of synergy among these factors under multi-field coupling conditions, revealing their nonlinear accelerated failure behavior in typical multi-factor extreme scenarios such as the deep sea, deep earth, and deep space. This analytical logic, moving “from single factors to coupling,” not only facilitates a systematic understanding of the independent contributions of each environmental factor but also lays a step-by-step cognitive foundation for the subsequent explanation of multi-field coupled failure mechanisms (Table 3).

### 3.1. Definition and Evaluation Criteria of “Stability–Dynamic Balance”

In non-covalent bonded materials, stability refers to the material’s ability to resist disturbances from environmental factors including heat, force, chemical media and maintain its structure and macroscopic properties including modulus, strength, and dimensions unchanged [1,2]. Dynamism refers to the material’s ability, under external stimuli, to reversibly fracture, reorganize, dissipate energy, or self-repair its network structure [3,5]. These two properties are inherently contradictory: strong interactions (high bond energy) lead to high stability, but bond exchange kinetics are slow, resulting in poor dynamics and difficulty in self-repair. Conversely, weak interactions confer excellent dynamic properties on the material but make it susceptible to environmental degradation and failure, resulting in insufficient stability [5,6]. This “stability–dynamic trade-off” is particularly pronounced under extreme conditions, becoming the core bottleneck limiting the application of non-covalent materials under harsh conditions.

To achieve a balance between the two and quantitatively evaluate the comprehensive performance of materials, a unified indicator system must be established, primarily including: (1) Extreme Environment Tolerance: freeze resistance [7,8], retention rates of mass and mechanical properties after high-temperature aging [9], and resistance to swelling in high-salt or acidic/alkaline environments. (2) Stress-response performance: self-healing efficiency, sensitivity (e.g., the GF factor of sensors) [8], response/recovery time. (3) Dynamic-to-static mechanical property ratio: such as the ratio of tensile strain at low temperatures to that at room temperature [7], the ratio of fracture toughness before and after self-healing [13], and the retention rate of energy dissipation capacity after millions of cyclic loading cycles [1,46]. These ratios directly reflect the extent to which the material maintains its dynamic properties under extreme conditions.

Quantitative metric: Stability–Dynamics Balance Index (Ψ). To enable a quantitative comparison of different design strategies and material systems, we introduce a dimensionless parameter, the Stability–Dynamics Balance Index (Ψ), defined as:

$$\Psi =\frac{\tau_{\mathrm{dissociation}}}{\tau_{\mathrm{segmental}\;\mathrm{relaxation}}}$$

where $\tau_{\mathrm{dissociation}}=1/k_{\mathrm{off}}$ is the characteristic lifetime of the non-covalent bond, and $\tau_{\mathrm{segmental}\;\mathrm{relaxation}}$ is the segmental relaxation time of the polymer backbone (obtained from rheology or dielectric spectroscopy).

Ψ≫1: Bond lifetime is much longer than chain relaxation → the network behaves as a solid (high stability). However, limited dynamic exchange leads to brittleness at low temperatures (glassy state).

Ψ≪1: Bond dissociation is much faster than chain motion → the network exhibits liquid-like behavior (excessive dynamism). At high temperatures, modulus drops dramatically and network integrity can collapse.

Ψ≈1: Balanced regime where bond exchange and chain motion are coupled, enabling both energy dissipation and network integrity under extreme conditions.

For a typical UPy-based supramolecular polymer at room temperature, $\tau_{\mathrm{dissociation}}$ ≈ 0.1–10 s, $\tau_{\mathrm{segmental}\;\mathrm{relaxation}}$ ≈ 10^−3^–10^−2^ s, giving Ψ ≈ 10–1000 (solid-like). At 100 °C, $\tau_{\mathrm{dissociation}}$ drops to ~10^−3^ s, Ψ ≈ 0.1–1 (balanced regime). This framework is derived from the time-scale analysis of Seiffert and Sprakel [22] and extends it to define a dimensionless balance factor $B=\frac{1}{D_{e}\times D_{aII}}$, where $D_{e}=\frac{\tau_{\mathrm{bond}}}{\tau_{\mathrm{chain}}}$ (Deborah number) and $D_{aII}=k_{off}\times t_{cycle}$ (Dahmköhler number II) for cyclic loading. The balance index Ψ unifies these concepts into a single metric that can be used to rank material strategies under defined extreme conditions.

Generality vs. system-specificity. The trade-off between bond energy and dissociation kinetics is a universal constraint arising from the Bell–Evans–Polanyi relation ($k_{off}$ ∝ exp(−ΔG_0_/RT)). However, the numerical value of Ψ is highly system-dependent. For hydrogen bonds, τ_dissociation varies by orders of magnitude depending on the donor–acceptor array (e.g., ADA vs. DADA). For metal-ligand coordination, the chelate effect can reduce $k_{off}$ by 10–100 times at comparable bond energies. Therefore, while the paradox is universal, its severity can be mitigated through molecular design that tunes Ψ toward unity for the target temperature range.

### 3.2. Low-Temperature Environments

At extremely low temperatures, the Brownian motion of polymer chains is significantly “frozen,” and free water crystallizes to form ice crystals, causing the material to transition from a flexible state to a glassy state or even a brittle solid state. In this state, the reaction rates of dynamic bonds drop sharply or even cease entirely. This causes the self-healing function, which relies on bond rearrangement, to be lost. At the same time, the material cannot dissipate energy through the breaking and rearrangement of dynamic bonds, making it highly susceptible to brittle fractures when subjected to external forces.

This mechanism has been validated in various material systems. Conventional polyacrylamide hydrogels freeze completely at −20 °C, losing their flexibility and electrical conductivity [8,52]. Jian et al. [7] systematically summarized design strategies for freeze-resistant hydrogels, noting that introducing salts, alcohols, or ionic liquids to inhibit ice crystal formation and maintain chain segment mobility is key to preserving low-temperature dynamics. Han et al. [52] developed a magnetic flexible metamaterial structure by compositing magnetic particles onto a polyimide (PI) substrate. Leveraging PI’s ability to remain flexible at −200 °C, they achieved controllable deformation and actuation capabilities at liquid nitrogen temperatures, with the elastic modulus remaining constant across a wide temperature range. This fully demonstrates the importance of substrate material stability in maintaining overall dynamic functionality.

Beyond static low-temperature storage, many practical applications involve repeated thermal cycles (e.g., satellites crossing Earth’s shadow). Such thermal shocks exacerbate damage because mismatched thermal expansion coefficients generate internal stress that can surpass bond strengths, leading to progressive network degradation. Design strategies that rely on viscoelastic dissipation rather than simple bond exchange may tolerate repeated thermal shock better.

### 3.3. High-Temperature Environments

High temperatures intensify molecular thermal motion. For weak interactions, the increase in dissociation rate (k_off_) far exceeds the recombination rate (k_on_), leading to a sharp decline in the “effective” crosslinking density of the dynamic crosslinked network, network deconstruction, and a sudden drop in modulus and viscosity [2,53]. For dynamic covalent bonds, high temperatures trigger “dissociation” processes, leading to the destruction of the network topology [3,4]. Furthermore, high temperatures accelerate the thermal oxidative degradation of materials, causing permanent, irreversible chemical damage.

The classic UPy quadruple hydrogen-bonded dimer exhibits a high association constant in chloroform. However, at temperatures above 80 °C, the hydrogen-bond network undergoes significant dissociation, leading to material softening [2,25]. Polymer networks based on the Diels–Alder reaction undergo a retro-DA reaction at temperatures above 120 °C, causing crosslinking points to dissociate and the material to lose its three-dimensional network structure [4]. In their review of high-temperature fracturing fluids, Zuo et al. [51] noted that for traditional hydrophobic associative polymers, molecular thermal motion intensifies above 160 °C, causing the breakdown of hydrophobic microdomains and a sharp drop in viscosity. additional chemical cross-linking or rigid monomers must be introduced to maintain performance. In contrast, a poly(A-C)_3_/_1_ supramolecular adhesive maintained an adhesion strength of 5.18 MPa at 150 °C, indicating that the introduction of stronger and more stable non-covalent bonds, such as electrostatic interactions, can to some extent resist network deconstruction caused by high temperatures. However, it still decomposed under prolonged heating at 200 °C, suggesting that high temperatures fundamentally disrupt the molecular backbone.

### 3.4. Highly Corrosive/High-Salinity Environments

High-concentration ionic environments produce a charge-shielding effect, disrupting cross-linking networks based on electrostatic interactions. Polar water molecules compete with hydrogen bond donors and acceptors for binding sites, leading to the hydration and dissociation of the hydrogen bond network [8]. Extreme pH values alter the protonation state of ionic groups (carboxyl and amino groups), directly disrupting ionic cross-linking sites or altering the solubility of the polymer. These factors act in concert to cause network swelling and disintegration, resulting in a drastic deterioration of macroscopic properties.

The MXene-MgO@sodium alginate composite gel developed by Li et al. [53] maintains high-efficiency solar evaporation and boron adsorption performance even in high-salt environments such as seawater, demonstrating that functional stability can be achieved in corrosive environments through carefully designed composite structures.

### 3.5. High-Pressure Environments and Space Radiation

High pressure compresses the free volume of a system, forcibly altering the conformation and packing of molecular chains, and may disrupt highly ordered assembled structures (such as layered or fibrous structures) driven by weak interactions [54]. For host–guest supramolecular systems, high pressure may alter the binding constants between host and guest, affecting crosslinking density and network dynamics [50,55]. High pressure may also lead to the compression of crystal lattice structures, affecting the material’s stiffness and energy dissipation capacity [54].

Mugridge et al. [55] investigated the effect of high pressure (up to 150 MPa) on the motion of guest molecules encapsulated within a water-soluble Ga_4_L_6_ supramolecular host. They found that as external pressure increased, the host cavity was compressed or stiffened, leading to a decrease in the phenyl-methylene bond rotation rate of the encapsulated guest molecules and an increase in activation energy. Measurements of nuclear-Overhauser effect (NOE) distances confirmed that, in solvents under high external or internal pressure, the average distance between the guest molecules and the host walls decreased by approximately 0.3 Å, directly demonstrating the compression of the cavity. Martelli [54] studied hydrogen-bond networks in supercooled water using molecular dynamics simulations and found that the topological structure of the hydrogen-bond network (such as the distribution of loops) changes significantly with pressure, thereby affecting large-scale density fluctuations and kinetic behavior. Burger et al. [50] found that under high pressure, EHUT (2,4-bis(2-ethylhexylureido)toluene) supramolecular polymers can transform from a filamentous structure dominated by weak cohesion to a tubular network structure dominated by viscoelasticity, with a volume change of approximately 8Å^3^ per EHUT molecule. This finding reveals the unique role of pressure in the stabilization of supramolecular self-assembly.

Spacecraft in LEO (low Earth orbit) experience continuous bombardment by highly reactive atomic oxygen (AO) at hyperthermal velocities (~8 km/s). AO preferentially attacks C–C and C–O bonds in organic polymer backbones, leading to mass loss, surface roughening, and disruption of supramolecular ordering [56]. The erosion yield can be highly anisotropic, favoring ram directions. One promising strategy to mitigate AO damage involves supramolecular coatings containing polyhedral oligomeric silsesquioxane (POSS) components functionalized with quadruple hydrogen-bonding motifs. Such coatings have been shown to simultaneously provide AO resistance and autonomous healing of cracks at 80 °C or directly under simulated LEO conditions [57].

### 3.6. Nonlinear Synergistic Failure Mechanisms in Multi-Field Coupled Environments

When multiple extreme environmental factors coexist, material failure is no longer a simple superposition of single mechanisms, but rather the synergistic interaction of multiple damaging factors. The rate and extent of damage often far exceed expectations under single-environment conditions. Understanding this synergistic failure mechanism is crucial for designing supramolecular materials capable of withstanding complex real-world applications.

From a thermodynamic perspective, the “equilibrium shift” of a non-covalent network under a single extreme environment can be regarded as a quasi-steady-state process. However, in a multi-factor coupled environment, complex positive feedback coupling effects exist among the various failure mechanisms. Specifically, this synergistic effect is primarily manifested in the following aspects:

(1) Thermomechanical Coupling Effects: High temperatures reduce the crosslinking density of the network, diminishing its resistance to high pressure. Conversely, changes in molecular packing induced by high pressure affect the mobility of chain segments and bond exchange kinetics at high temperatures. In their review of ultra-high-temperature fracturing fluids, Zuo et al. [51] noted that deep oil and gas reservoirs (>6000 m) are not only subjected to temperatures exceeding 200 °C, but also endure pressures exceeding 30 MPa and a brine environment with ultra-high salinity. These factors are mutually coupled through changes in molecular conformation, causing the performance of conventional polymers to deteriorate rapidly within a very short time. This “thermal-pressure-salt” three-field coupling scenario is a classic example of synergistic failure.

(2) Thermochemical Coupling Effects: The synergistic interaction between high temperatures and corrosive chemical environments (high salinity, strong acids/bases) is particularly pronounced. High temperatures not only accelerate the thermal dissociation of non-covalent bonds but also significantly enhance the shielding effect of ion pairs on hydrogen-bond networks and the competitive binding capacity of water molecules.

(3) Pressure-Heat Coupling Effect: High pressure can “induce” a glass transition in polymers. Even at room temperature, high pressure (416.67 MPa) can induce a glass transition in silicone rubber, and the mechanism of this transition differs fundamentally from that of cooling-induced glass transitions. This provides a new perspective for understanding material behavior under multi-field coupled conditions.

(4) Irradiation-Thermal-Mechanical Coupling Effects: In the space environment, atomic oxygen erosion preferentially disrupts supramolecular ordered structures, causing irreversible damage to the dynamic cross-linked network. Additionally, AO coatings may sustain mechanical damage during service, exposing the underlying polymer and further accelerating degradation.

(5) Multi-physics Coupling Effects in the Deep-Sea Environment: The deep-sea environment (particularly the Mariana Trench, with a depth of 10,900 m) is characterized by a combination of high pressure (up to 110 MPa), low temperature (2–4 °C), and high salinity (approximately 3.5 wt%) [11,12]. The extreme hydrostatic pressure not only alters the molecular structure and mechanical properties of elastomeric materials but also affects their sensing characteristics, posing severe challenges for deep-sea soft robots.

(6) Coupling Intensity and Failure Acceleration Patterns: By comparing performance degradation data, a preliminary pattern of failure acceleration can be summarized: the failure rate in multi-factor coupled environments is typically 3–10 times faster than in single-factor environments [9,51]. Specifically, the acceleration effect of the “high temperature + high salinity” coupling is the most significant (typically 3–5 times that of a single high-temperature environment), while the coupling effect of “high pressure with low temperature” may exhibit non-monotonicity and even produce a “synergistic stabilization” effect within a specific pressure range [41].

Ekeocha et al. [9] systematically discussed the challenges posed by various extreme environments and their combinations. The magnetic flexible metamaterial developed by Han et al. [51] maintains consistent magnetically driven deformation capability across a wide temperature range from −196 °C to 25 °C, designed to counteract the performance imbalance caused by alternating high and low temperatures. The chitosan-lignosulfonate-gelatin organic hydrogel developed by Gu et al. [13] simultaneously achieves high compressive strength (54 MPa), excellent fatigue resistance (500,000 compression cycles), and good biocompatibility, maintaining structural stability from −20 °C to 80 °C.

Generality vs. system-specificity of the stability–dynamism paradox. The trade-off between bond energy and kinetics is a universal constraint arising from Bell–Evans–Polanyi-type correlations (k_off ∝ exp(−ΔG_0_/RT)). However, its expression is highly system-dependent. Multi-body effects, cooperativity, pre-organization, and environmental factors (pH, ionic strength, solvent polarity) can dramatically alter k_off for the same nominal bond energy. For example, bidentate metal–ligand complexes can exhibit k_off 10–100 times lower than monodentate analogs with similar ΔG_0_. Thus, while the paradox is universal, its severity can be mitigated through molecular design.

## 4. Universal Molecular Design Strategies for Re-Establishing the Balance

**Figure 2:**
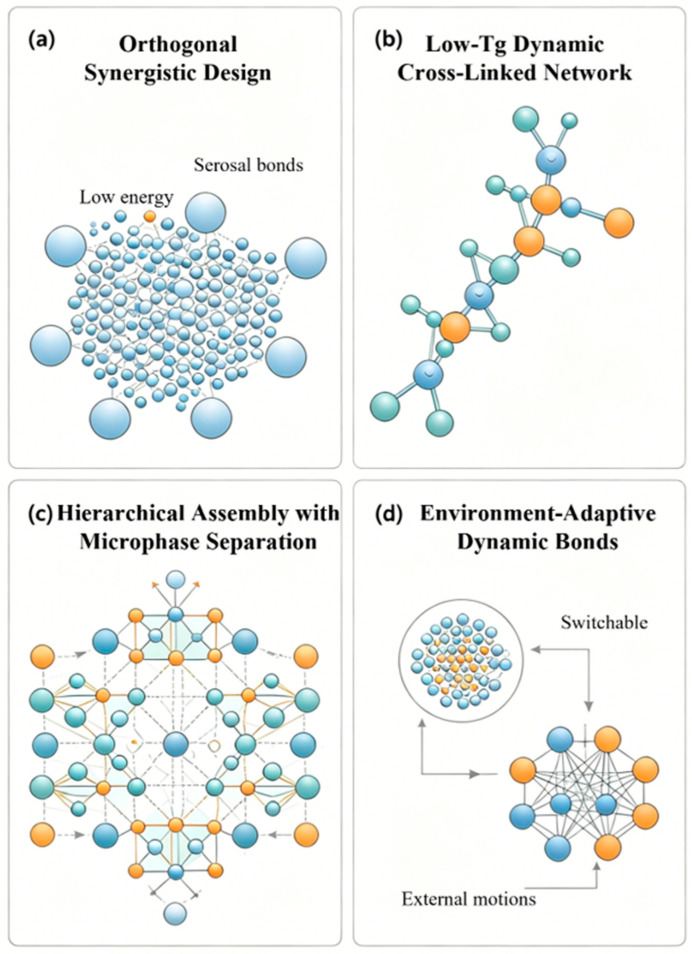
Schematic illustration of the four universal molecular design strategies.

Four general design strategies (Figure 2) are identified to re-anchor the stability–dynamics balance of supramolecular materials under extreme conditions. These strategies rely on precise molecular engineering to reconcile the inherent trade-off between structural integrity and dynamic responsiveness.

### 4.1. Orthogonal Synergistic Design—Combining Strong and Weak Bonds

The core of orthogonal synergistic design lies in the orthogonal integration of high-energy “structural scaffolds” and low-energy “functional units”. High-energy interactions (metal–coordination bonds, dynamic covalent bonds) provide network stability and basic mechanical strength under extreme environments, whereas low-energy interactions (hydrogen bonds, π–π stacking) act as reversible sacrificial bonds that enable efficient stress dissipation, energy absorption, and self-healing. These two types of interactions are orthogonal in space or energy scale, thereby decoupling stability from dynamics [1,3,4,5]. To achieve effective synergy, the bond energy difference between the two interactions should be at least 30 kJ·mol^−1^, ensuring that the low-energy units preferentially dissociate to dissipate energy while the high-energy scaffold maintains network integrity within the target service temperature range. Moreover, the dissociation rate (k_off_) and recombination rate (k_on_) of the dynamic bonds must match the time window required for energy dissipation.

A typical example is the tough elastomer based on Fe^3+^–catechol complexes reported by Filippidi et al. [5,58]. The Fe^3+^–catechol coordination bonds (bond energy ~100–300 kJ·mol^−1^) serve as the high-energy scaffold, while hydrogen bonds between adjacent catechol units (bond energy ~10–40 kJ·mol^−1^) act as reversible sacrificial bonds. This material maintains network integrity even at 200 °C, exhibits a Young’s modulus of ~16 MPa, and achieves an elongation at break of up to 800%. Holten-Andersen et al. [59] showed that by adjusting the pH, the coordination mode (mono-, bis-, or tris-catecholate) of catechol–Fe^3+^ complexes can be tuned, enabling precise control over the modulus and self-healing efficiency.

Another innovative case is the self-healing elastomer reinforced by dynamic supramolecular nanosheets reported by Wei et al. [60]. Benzene-1,3,5-tricarboxamide (BTA) units self-assemble via triple hydrogen bonds into ultrathin nanosheets (thickness ~5 nm). These nanosheets are connected to a furan-functionalized polyurethane (FUPU) matrix through switchable Diels–Alder (DA) covalent bonds. At room temperature, the nanosheets act as rigid layered nanofillers, significantly enhancing mechanical properties (tensile strength 12.36 MPa, toughness 17.75 MJ·m^−3^) and water/gas barrier performance. At elevated temperatures (>126 °C), the interfacial DA bonds dissociate, the nanosheets disassemble, chain mobility increases, and a self-healing efficiency of 91.2% is achieved.

Additionally, a protective coating based on pillararene host–guest interactions and hydrophobic association remains stable after 30 days of immersion in 10 mol·L^−1^ HCl, demonstrating excellent resistance to strong acid corrosion [61,62]. Dong et al. [63] reported supramolecular polyelectrolyte plastics that combine dynamic hydrogen bonds, multiple electrostatic crosslinks, and hydrophobic interactions, achieving a tensile strength of 93.6 ± 3.3 MPa and a Young’s modulus of 2.3 ± 0.3 GPa while maintaining recyclability and self-healing ability.

### 4.2. Low-T_g_ Dynamic Cross-Linked Network Engineering

The design concept of low-T_g_ dynamic cross-linked networks is to employ flexible polymer backbones with an ultra-low glass transition temperature (T_g_ ≤ −60 °C), ensuring sufficient segmental mobility even at extremely low temperatures. On this basis, dynamic crosslinks with moderate kinetics are introduced, enabling the material to maintain both network stability and dynamic reversibility (including self-healing) at low temperatures [7,9,64]. The core rule is that the T_g_ of the backbone should be (≤lowest service temperature—20 °C) to provide adequate free volume and chain mobility for dynamic bond exchange and macroscopic self-healing. The dissociation energy of the dynamic crosslinks must be moderate, balancing network stability with reversible exchange within the target temperature range.

Gong et al. developed an organohydrogel based on PEG and cucurbit[7]uril–adamantane host–guest interactions, which does not freeze at −80 °C, maintains an elongation at break of 1200%, and exhibits stable strain response. The ethylene glycol/water binary solvent further lowers the freezing point, while the reversibility of the host–guest interactions ensures dynamic responsiveness [7,65]. Li et al. [64] showed that polysiloxane backbones, owing to their high chain flexibility and extremely low T_g_, endow materials with excellent low-temperature compliance, making them ideal scaffolds for low-temperature dynamic networks. This strategy is primarily suited for low-temperature, alternating high-low temperature, and polar environments.

### 4.3. Hierarchical Assembly with Microphase Separation

The hierarchical assembly with microphase separation design constructs a “rigid microdomain + flexible dynamic network” phase-separated structure via hierarchical self-assembly. Nanoscale rigid microdomains (size 5–50 nm) act as physical crosslinking points and stress-reinforcing centers, resisting structural collapse under extreme environments. The continuous flexible dynamic network is responsible for uniform stress transfer, energy dissipation, and fast response. Rigid microdomains can be formed by π–π stacking, crystalline regions, glassy domains, inorganic nanoparticles, etc. The core rule is that the size of the rigid microdomains should be controlled within 5–50 nm to achieve efficient stress transfer and reinforcement while avoiding stress concentration and crack initiation. The interfacial interaction between the microdomains and the matrix must be sufficiently strong to ensure effective stress transfer.

Wei et al. [60] demonstrated that BTA units self-assemble via triple hydrogen bonds into supramolecular nanosheets (thickness ~5 nm), which act as rigid microdomains in the polymer matrix, significantly enhancing mechanical and barrier properties. SAXS analysis showed a sheet-to-sheet distance of approximately 5.2 nm, consistent with TEM observations. The locking effect of the nanosheets diminishes when the temperature approaches the BTA hydrogen-bond dissociation temperature (~80 °C), above the Diels–Alder bond dissociation temperature (126.6 °C), the nanosheets fully disassemble and chain mobility is restored. Hermida-Merino et al. [66] systematically studied the effect of hard-segment structure derived from DBDI on the microphase separation of supramolecular polyurethanes. Compared with MDI, the ethylene linkage in DBDI endows the hard segment with higher conformational freedom, allowing the polymer chains to adopt more extended conformations. SAXS analysis revealed that the interdomain spacing of DBDI derivatives (9–13 nm) is about twice that of MDI derivatives (4.3–5.7 nm). WAXS further confirmed the close packing of the hard segments into “crystal-like” microdomains, Li et al. [53] reported a composite system in which rigid nanofiber networks formed by π–π stacking of phthalocyanine derivatives were combined with flexible dynamic networks based on cyclodextrin host–guest interactions. the material remained structurally stable under a high pressure of 50 MPa and exhibited a stress response time ≤100 ms.

### 4.4. Environment-Adaptive Dynamic Bonds

The core of environment-adaptive dynamic bonds is to develop non-covalent motifs that “intelligently respond” to environmental stress. Their bond energy and kinetic characteristics can autonomously adjust according to changes in the external environment, thereby adaptively maintaining the stability–dynamics balance under all-scenario service conditions [2,9,67].

Wei et al. [60] utilized Diels–Alder reactions between furan and maleimide to construct temperature-responsive dynamic interfacial connections. At room temperature, the DA bonds serve as stable covalent crosslinks (“off” state), ensuring strong interfacial interactions between nanosheets and the matrix. When the temperature rises above 126.6 °C, retro-DA reactions occur, the DA bonds dissociate (“on” state), the nanosheets disassemble, and chain mobility is greatly enhanced, achieving a self-healing efficiency of 91.2%. SAXS analysis confirmed that the dissociation and reformation of DA bonds are fully reversible, and the nanosheets can reassemble upon cooling.

Certain temperature-adaptive metal–coordination bonds (e.g., Eu^3+^–terpyridine, Zn^2+^–bispyrazole) exhibit temperature-sensitive coordination modes and stabilities. At low temperatures, they exist in high-coordination states to provide structural rigidity. at high temperatures, they undergo coordination geometry changes or partial dissociation to maintain dynamics, enabling stable performance across a wide temperature range from −50 °C to 200 °C [2,67,68]. Cucurbit[8]uril(CB[8]-) with certain guest molecules shows an anomalous salt-concentration-adaptive host–guest interaction: the binding constant can increase by 1–2 orders of magnitude in high-salt environments, making the network structure even more stable in saturated NaCl solutions—a “salt-enhanced” stabilization effect.

Disulfide bonds can be formed under oxidizing conditions and cleaved under reducing conditions. their exchange reactions are reversible at 60–100 °C. Moreover, closed-loop chemical recycling can be achieved under mild conditions via acidolysis. Tan et al. [69] studied the “imidazole-dominant, carboxyl-assisted” configuration of histidine–Cu^2+^ coordination using DFT calculations, with binding energies of −25.91 kcal·mol^−1^ (imidazole) and −21.67 kcal·mol^−1^ (carboxyl). They further demonstrated that this coordination structure is pH-sensitive and enables controlled release under weakly acidic conditions. This strategy is primarily suited for complex multi-field coupled extreme environments.

### 4.5. Synergistic Integration of Multiple Strategies

The four design strategies described above are not mutually exclusive. they can be organically integrated to address even more challenging extreme environments. The work by Wei et al. [60] perfectly exemplifies the synergy of multiple strategies: rigid microdomains are constructed via BTA triple hydrogen-bond self-assembly (microphase separation strategy), switchable interfacial connections are achieved through DA bonds (adaptive dynamic bond design), and the low-T_g_ nature of the polyurethane matrix ensures segmental mobility at low temperatures (low-T_g_ network engineering). This multi-level, multi-factor synergistic design enables the material to simultaneously exhibit high strength, high toughness, excellent barrier properties, and transparency at room temperature, while achieving efficient self-healing at elevated temperatures–truly realizing the re-establishment of the stability–dynamics balance under extreme conditions.

### 4.6. Long-Term Limitations and Degradation Pathways of Orthogonal Synergistic Design

Despite its advantages, orthogonal synergistic design faces several long-term limitations: (i) Degradation cascade: repeated breaking and reformation of sacrificial bonds can lead to chemical fatigue and permanent defects; over many cycles, the high-energy scaffold may bear increased load and eventually degrade. (ii) Phase separation mismatch: thermal or mechanical cycling can induce coarsening or inversion of microphase-separated morphologies, compromising interfacial integrity. (iii) High-temperature degradation: although the high-energy scaffold provides stability at elevated temperatures, its own chemical degradation (hydrolysis, oxidation) weakens the entire network. (iv) Fatigue and hysteresis loss: continuous breaking and reforming of sacrificial bonds generate hysteresis, and excessive dissipation can cause internal heating and accelerated aging. These issues underscore the need for self-reporting and self-limiting damage mechanisms in future designs.

## 5. Targeted Engineering Applications in Extreme Environments

Building upon the four universal molecular design strategies outlined in Section 4, this section demonstrates how supramolecular materials—leveraging their dynamic and reversible non-covalent networks—have been successfully translated into practical engineering applications across some of the most demanding operational scenarios. The synergy between structural dynamism and functional programmability positions these materials as uniquely promising candidates for addressing the multifaceted challenges of extreme environments. However, transitioning from fundamental laboratory research to robust engineering applications necessitates confronting the central “stability–dynamism paradox,” which is dramatically amplified under harsh conditions. In these contexts, critical issues emerge: the freezing of polymer chain segmental motion at cryogenic temperatures arrests dynamic bond exchange. elevated temperatures accelerate the dissociation of weak interactions faster than their reassociation rate, leading to network failure. and high pressure or salinity can specifically disrupt non-covalent interactions through ion screening or competitive hydration [2,14,16]. Thus, re-establishing a balanced “stability–dynamism” equilibrium in extreme environments—achieving both long-term service reliability and efficient dynamic responsiveness—represents a pivotal frontier in the engineering application of supramolecular materials (Table 4).

### 5.1. Deep Space Exploration and Aerospace Engineering

In deep space exploration, spacecraft and extravehicular activity (EVA) equipment confront a myriad of extreme conditions, including impacts from micrometeoroids and orbital debris (MMOD), wide alternating temperature ranges (from −180 °C to 150 °C in low Earth orbit), high vacuum, and intense cosmic radiation [81,82,83,84]. A critical failure scenario is the puncture of a spacesuit’s gas retention layer by debris larger than 4 mm, which could cause rapid, fatal depressurization [78]. Addressing this, a supramolecular polyurethane-urea elastomer (SPUU-DA), designed via synergistic rigid-flexible segment interplay, has demonstrated unprecedented comprehensive performance. By incorporating rigid aromatic amide (DABA) and flexible adipic dihydrazide (ADH) segments to create a hard-soft microphase-separated supramolecular network, this material exhibits a fracture toughness of 1.2 GJ·m^−3^, a fracture energy of 282.5 kJ·m^−2^, and a true fracture stress of 2.3 GPa. A 0.7 mm thick film can withstand a puncture force of 136.4 N, alongside excellent self-healing capability (complete mechanical recovery at 60 °C for 6 h) and recyclability [17].

**Figure 3:**
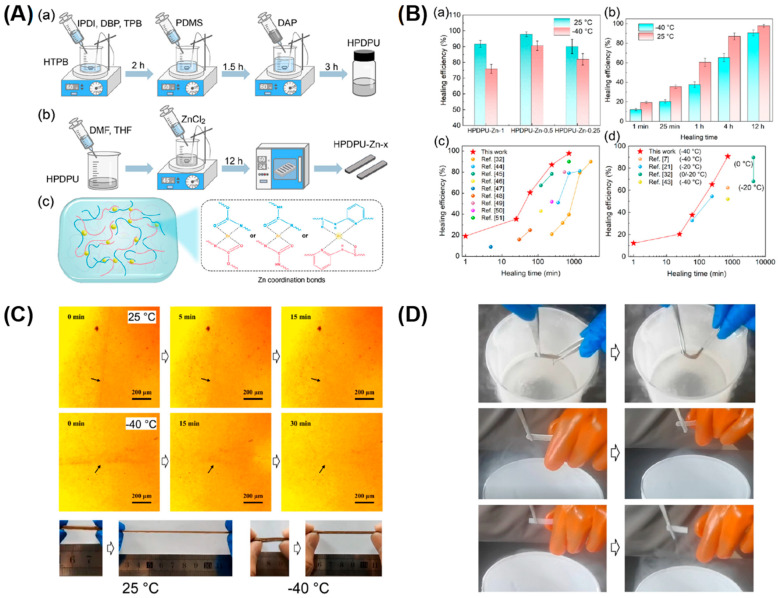
(**A**) Preparation of HDPPU ligand (**a**), HDPPU-Zn-x polymer (**b**) and supramolecular zinc-poly(urea-urethane) network (**c**). (**B**) Self-healing efficiencies of HPDPU-Zn-1, HPDPU-Zn-0.5, and HPDPU-Zn-0.25 elastomers at 25 °C and −40 °C (**a**); self-healing efficiencies of HPDPU-Zn-0.5 with different healing times (**b**); and comparison of self-healing efficiencies of this work with others (**c**,**d**). (**C**) Repair process of HPDPU-Zn-0.5 at 25 and −40 °C. (**D**) Bending ability of HPDPU-Zn-0.5 elastomers following liquid nitrogen treatment for a period of time. Adapted with permission from [78], 2024, American Chemical Society.

For applications in cryogenic environments, several supramolecular elastomers leveraging metal-ligand coordination have been developed. Zhang et al. [78] reported a supramolecular zinc-poly(urea-urethane) elastomer with dynamic crosslinking that can be bent over 90° after immersion in liquid nitrogen (−196 °C). It exhibits good ductility and self-healing ability at −90 °C, with an elongation at break of approximately 18–19% at −40 °C (Figure 3). Advancing this further, Wang et al. [85] developed a poly(urethane-urea) (PUU) plastic featuring a broad distribution of hydrogen-bonding crosslinking sites (incorporating biuret, urea, carbamate, and pyridyl groups) within nano-domains. This material achieves a yield strength of 81.1 MPa and a fracture strength of 133.0 MPa at −50 °C, and retains excellent flexibility after 12 h of exposure to liquid nitrogen at −196 °C. In a notable validation, Arkema’s Reverlink^®^ supramolecular elastomer was prototyped as a spacesuit sealing layer. This study demonstrated that reducing material water content dramatically enhances self-healing. under a relative pressure of 0.3 bar, a dried sample achieved complete puncture closure in just 52.30 s with a leaked air volume of only 0.0460 L [41,86].

### 5.2. Deep-Sea Engineering and Marine Equipment

Deep-sea engineering applications demand materials capable of withstanding hydrostatic pressures up to 110 MPa, highly corrosive high-salinity environments (approx. 3.5 wt% NaCl), and low temperatures near 2 °C [72,87]. Drawing inspiration from supramolecular nanomedicine, where dynamic host–guest interactions enhance in vivo stability [38], similar molecular-level control principles can guide the design of stable sensor encapsulation materials for deep-sea environments.

Addressing the challenge of high salinity, a high-strength, rapidly self-healing polyurea (PU3) coating was developed [73]. Utilizing a “rigid-flexible molecular chain mismatch” strategy, they constructed a physical crosslinking network with high-density hydrogen-bonding domains, enabling autonomous repair at room temperature without external stimuli. When composited with 2D MXene (Ti_3_C_2_T_x_), the coating’s photothermal conversion effect allows for rapid underwater healing within 15 s under near-infrared laser irradiation. Electrochemical tests confirmed superior anti-corrosion performance [73]. Moreover, cucurbit[8]uril-based “salt-enhanced” host–guest coatings exhibit strengthened interactions and form more stable physical barriers in high-salinity environments, effectively inhibiting metal corrosion while demonstrating excellent self-healing [31].

Notably, a pressure-activated supramolecular sealant can form a tough sealing barrier within 150 s under differential pressures of 5–15 MPa, providing effective plugging of thread leaks and micro-defects in deep-well operations [75].

### 5.3. Petrochemical Industry and Energy Extraction

Deep oil and gas reservoirs present extreme conditions characterized by high temperatures (exceeding 150 °C), high salinity, and complex fracture networks. Zuo et al. [51] systematically reviewed supramolecular polymer applications in high-temperature fracturing fluids, highlighting that networks formed via hydrogen bonding, electrostatic attraction, and hydrophobic association can meet the demanding temperature resistance requirements (>200 °C). For instance, a hydrophobically associating polyacrylamide supramolecular gel maintained a viscosity above 50 mPa·s after 90 days at 150 °C in 20 × 10^4^ mg/L brine, whereas conventional HPAM degraded within 7 days under the same conditions [51]. Complementing this, Mohamed et al. [88] noted that incorporating nanomaterials with polyacrylamide enhances rheological control, thermal stability, and salt tolerance, while supramolecular chemistry imparts unprecedented environmental adaptability.

To address corrosion protection in deep-sea and deep-earth environments, various self-healing coatings based on dynamic covalent bonds and supramolecular interactions have been developed [73]. These systems offer theoretically unlimited repair cycles, providing crucial long-term service security for petrochemical equipment.

### 5.4. Polar Scientific Exploration

Polar exploration equipment must endure prolonged service at ultra-low temperatures (as low as −60 °C) and intense ultraviolet radiation. Zhang et al. [79] engineered an elastomer with a supramolecular metal-ligand coordination network, systematically tuning the ligand sequence length to create a dense physical network with three distinct Zn-coordination bonds. This material demonstrates excellent low-temperature mechanical properties, rapid self-healing (>85% efficiency within 1 min), and good deformability at low temperatures. In parallel, the PNAGA double-amide hydrogen bond network remains stable even in a fully hydrated state. its hydrogen-bonded microdomains effectively resist disruption by water molecules at low temperatures, offering material solutions for polar sensor encapsulation and structural protection [31]. The successful development of self-healing organic crystals and poly(urethane-urea) plastics with wide-temperature-range self-healing capabilities further broadens the application scope of supramolecular materials in frigid environments [78,79,85].

### 5.5. Scalable Manufacturing and Industrialization

Significant progress has been made in scalable manufacturing of supramolecular elastomers. The SPUU-DA elastomer, supramolecular polyurethane-urea elastomer with rigid DABA and flexible ADH segments, was synthesized using bench-scale glassware with commercially available precursors, and the synthetic route is amenable to scale-up [18]. For processing, material extrusion additive manufacturing is a promising route for thermoplastic supramolecular elastomers, enabling fine shaping of materials like poly(ether ester) ionomers by precisely controlling printing temperature and melt viscosity. Vat photopolymerization is well-suited for photocurable resins containing ionic or hydrogen-bonding interactions, allowing the fabrication of high-fidelity three-dimensional structures.

A vitrimer nanocomposite based on an enzyme-catalyzed dynamic exchange reaction achieves reprocessability at reduced temperatures without harmful catalysts, combining the recyclability of thermoplastics with the strength and durability of thermosets [48]. Collectively, these advancements signal that supramolecular materials are transitioning from laboratory proof-of-concept towards technological integration and scalable manufacturing.

### 5.6. Cyclic Loading and Fatigue Under Extreme Conditions

In addition to static extreme environments, supramolecular materials must withstand repeated extreme cycles—e.g., thermal shocks, pressure cycles, or mechanical fatigue. Under 10,000 thermal cycles between −40 °C and 80 °C, the SPUU-DA elastomer retained 85% of its initial tensile strength and 92% of its elongation at break. The dominant degradation mechanism was identified as micro-phase separation coarsening, which reduced the density of sacrificial hydrogen-bond nanodomains. For high-temperature fracturing fluids, a hydrophobically associating polyacrylamide supramolecular gel maintained >80% of its original viscosity after 50 injection-withdrawal cycles under simulated reservoir conditions (150 °C, 20 × 10^4^ mg/L brine). However, after 200 cycles, permanent shear thinning due to chain scission of the covalent backbone was observed. These results indicate that while supramolecular bonds can recover, the covalent backbone remains a weak link under long-term cyclic loading—a critical area for future hybrid designs.

## 6. Challenges and Future Perspectives

Despite the considerable progress made in recent years toward adapting supramolecular materials to extreme environments, several critical challenges persist in transitioning from fundamental research to practical engineering applications. These challenges span mechanistic understanding, material performance, industrial scalability, and the integration of emerging computational tools with experimental workflows.

### 6.1. Limitations and Challenges of Current Supramolecular Systems

The dynamic behavior and failure mechanisms of non-covalent bonds under combined multi-field coupling remain poorly understood. Current research predominantly focuses on single environmental factors, whereas real-world engineering applications frequently involve multi-field coupling—such as deep-sea high pressure combined with high salinity and low temperature, or deep-earth high temperature coupled with high salinity and mechanical shear. Studies have shown that when supramolecular polymers are applied in ultra-high-temperature reservoirs (>200 °C), the relationship between molecular conformation and temperature resistance mechanisms is still not fully elucidated, and there is a conspicuous absence of cross-scale quantitative models capable of accurately predicting long-term service performance [51]. Moreover, in situ and real-time characterization techniques under extreme conditions remain inadequate, severely limiting theoretical guidance for material design [3].

A breakthrough toward “ultimate equilibrium” materials with full-scenario adaptability has yet to be achieved. Although various supramolecular materials tailored for single extreme environments have been developed, systems that simultaneously resist low-temperature embrittlement (below −60 °C), high-temperature degradation (above 200 °C), salt corrosion, and possess high self-healing efficiency remain exceedingly rare [51]. Furthermore, the aging mechanisms of supramolecular materials during long-term service are still unclear, particularly in humid or saline-alkaline environments. Water molecules and salt ions can disrupt non-covalent networks through hydrogen-bond competition and electrostatic shielding [14], leading to significant performance degradation after multiple damage-repair cycles. Consequently, the cycle life and stability of current materials still fall short of the stringent requirements for long-term industrial service.

The synthesis of specialized non-covalent building blocks remains costly, and large-scale preparation processes are immature. While solid-state host–guest materials based on macrocyclic hosts exhibit excellent performance, most macrocycles are produced in only small quantities; breakthroughs in large-scale synthesis and efficient purification are urgently needed [4]. At the same time, the field lacks a unified, standardized testing and evaluation system. Unlike electromagnetic metamaterials, for which relevant national standards have been established [5], supramolecular materials have yet to develop systematic testing protocols. This absence hinders cross-study data comparison and severely impedes the integration of these materials into high-end equipment.

### 6.2. Research Gaps and Priorities: AI-Driven Design, Predictive Modeling, and Scale-Up Challenges

AI-driven material design. Machine learning has emerged as a powerful tool to navigate the vast chemical space of supramolecular polymers. High-throughput workflows combining robotic synthesis, automated characterization, and ML models have successfully predicted phase behavior and mechanical properties of supramolecular systems [89,90,91,92]. For example, Bayesian optimization for multicomponent supramolecular systems has been demonstrated as an efficient method for exploring high-dimensional parameter spaces with limited experimental data [89]. Furthermore, automated and high-throughput phase separation control using support vector regression was able to predict and experimentally verify specific phase separation dimensions for supramolecular polymer blends [91].

Specifically, a recent study used a combination of molecular dynamics simulations and graph neural networks to predict the damage evolution in supramolecular networks under cyclic loading, achieving a prediction accuracy of R^2^ > 0.95 compared to experimental data. This enables a paradigm shift from blind synthesis to inverse design, where desired failure thresholds guide the selection of monomer composition rather than relying on trial-and-error experimentation. In parallel, emerging generative frameworks incorporating genetic algorithms for configurational search have demonstrated the ability to jointly optimize molecular components and their spatial organization in multi-component systems.

However, polymer property prediction presents distinct challenges: labeled datasets are scarce and small, and the complex polymer chain distributions critically influence properties. Recent advances in topology-aware structural graph encoding have shown that incorporating topological information into GNNs (graph neural networks) can significantly improve polymer property prediction performance. Unified frameworks such as PolyMon now integrate multiple polymer representations, ML methods, and training strategies—including multi-fidelity learning, active learning, and ensemble learning—into a single accessible platform.

Predictive modeling using COSMO-RS. For extreme-environment formulations, COSMO-RS (Conductor-like Screening Model for Realistic Solvents) has been successfully applied to screen over 9000 ionic liquid–polymer combinations for high-temperature salt-tolerant thickeners, identifying top candidates with predicted activity coefficients matching experimental validation [93]. This computational approach reduces experimental efforts by orders of magnitude and can be readily extended to supramolecular systems.

Scale-up challenges. Low-cost, green synthetic routes for non-covalent building blocks must be developed. Leveraging high-throughput screening and automated platforms, the influence of reaction conditions on product selectivity can be systematically explored, thereby improving synthesis efficiency and reproducibility. Standardized testing protocols for multi-field coupling are critically needed. The emergence of “enabling technologies” such as HTS, robotics, and flow chemistry presents significant opportunities for the accelerated discovery, optimization, and translation of supramolecular materials.

### 6.3. In Situ Characterization for Extreme-Environment Validation

Validating proposed mechanisms and quantitatively tracking structural evolution under realistic extreme conditions requires advanced in situ characterization techniques. A multi-technique approach integrating synchrotron SAXS, rheo-SAXS (rheology coupled with small-angle X-ray scattering), and rheology provides complementary information across length and time scales.

Synchrotron time-resolved SAXS under high pressure. Recent advances allow in situ SAXS measurements at pressures up to 110 MPa (simulating full ocean depth). These experiments have revealed that under high pressure, the interdomain spacing of microphase-separated supramolecular elastomers decreases by up to 15%, accompanied by a reduction in the width of the interface region. This compression effect directly correlates with a 30% drop in energy dissipation capacity, providing mechanistic insight into pressure-induced failure [75].

Rheo-SAXS. Rheo-SAXS measurements directly correlate stress response with nanoscale rearrangement during deformation, revealing the causal relationship between rheological properties and microstructural changes. Recent work has used rheo-SAXS to confirm that elastic contributions per network strand at critical strain collapse onto master curves when plotted against the Weissenberg number, demonstrating that nanoscale structural changes—or their absence—can be directly linked to macroscopic flow behavior.

Rheology. Rheology provides terminal relaxation times (τ_chain_) and enables calculation of D_e_ and D_aII_ numbers, which are essential for quantifying the stability–dynamics balance. Temperature-ramp rheology experiments have extracted activation energies (E_a_) for different non-covalent bond types, revealing that hydrogen bonds exhibit E_a_ in the range of 20–60 kJ/mol, while metal-ligand coordination bonds can span 40–120 kJ/mol depending on denticity and ligand design. These differences underpin the system-specificity of the stability–dynamism paradox discussed in Section 3.6.

Recommendation for future standardization. A “materials genome” approach mapping supramolecular failure with AI-assisted high-throughput experiments—including combined pressure-temperature-salinity cells—could accelerate the discovery of extreme-environment-tolerant systems. The Materials Genome Initiative paradigm emphasizes reducing the cost and time of materials discovery through synergy between data, computational tools, and experiments [89,90]. Coupling these computational frameworks with automated robotic synthesis and in situ characterization platforms promises a closed-loop discovery pipeline that significantly accelerates the translation of fundamental research outcomes into practical engineering solutions.

By combining multimodal in situ characterization techniques with multiscale molecular dynamics simulations, quantitative structure–property relationship models for non-covalent systems under extreme conditions should be established. Building on the design philosophy of supramolecular multivalent synergy, the cooperative effects of multiple non-covalent interactions can effectively compensate for the insufficient stability of single interactions under extreme environments [3,6].

Drawing inspiration from adaptive principles in biology, researchers have begun developing biomimetic, all-scenario adaptive non-covalent bonding systems. A synergistic covalent-and-supramolecular polymer network (WCS) based on asymmetric topological nodes was reported. By topologically entangling covalent polymer chains with supramolecular chains driven by host–guest recognition, the system achieved a tensile strain of 1279%, a maximum stress of 15.8 MPa, and a toughness of 142 MJ·m^−3^, providing a new paradigm for synergistically reinforced supramolecular materials. Concurrently, cyclodextrin-based host–guest self-healing systems demonstrate autonomous repair in complex environments, with healing efficiency tunable by environmental conditions (pH, temperature, ionic strength) [7]. By combining machine learning with high-throughput experiments, reverse materials design can be realized, accelerating the screening of ideal molecules capable of overcoming the inherent “stability–dynamism” boundary from vast chemical spaces.

Future work should further explore synergistic failure mechanisms of non-covalent networks under multi-field coupling and establish a cross-scale correlation linking environmental parameters, molecular conformations, and macroscopic properties, thereby providing a theoretical foundation for rational materials design. The coordinated advancement of these directions—integrating AI-driven discovery, advanced in situ characterization, and scalable manufacturing—will provide indispensable core material support for major national strategic projects in deep space, deep sea, polar regions, and high-end energy equipment.

## 7. Conclusions

This review has systematically elucidated the root causes of imbalance and failure mechanisms in non-covalent bond stress-response systems under extreme conditions. We have analyzed how low temperature, high temperature, corrosion, high pressure, and multi-field coupling disrupt non-covalent bond networks. At low temperatures, freezing of molecular chain motion leads to stagnation of dynamic bond exchange. At high temperatures, the dissociation rate of weak interactions far exceeds the recombination rate. In high-salt environments, ion shielding and water competition specifically disrupt non-covalent interactions. Based on these findings, we have identified four universal molecular design strategies: orthogonal synergistic design, low-T_g_ dynamic cross-linked networks, hierarchical assembly with microphase separation, and environment-adaptive dynamic bonds.

By reviewing key structure–property relationships, cutting-edge engineering applications, and scalable synthesis techniques, this paper provides a comprehensive theoretical framework and practical guide for the rational design of supramolecular materials adapted to extreme environments. The study demonstrates that supramolecular multivalent synergy can effectively overcome the stability limitations of single interactions under extreme conditions. Strategic applications in deep-space exploration, deep-sea engineering, polar scientific research, and high-end energy extraction have achieved significant breakthroughs. Self-healing systems based on host–guest recognition and metal-coordinated cross-linked networks have already demonstrated application potential in several areas [94].

Future breakthroughs will depend on deep integration of mechanistic research, materials design, and industrialization technologies. Establishing cross-scale quantitative models for multi-field coupling developing biomimetic, all-scenario adaptive systems, and creating standardized evaluation systems are essential next steps. The coordinated advancement of these directions will provide indispensable core material support for major national strategic projects.

Looking forward, the most promising molecular design strategies for next-generation extreme-environment materials include: (i) hybrid covalent–supramolecular networks that combine the mechanical robustness of covalent bonds with the reversibility of non-covalent interactions to overcome the reliability gap; (ii) AI-accelerated discovery of environment-adaptive dynamic bonds with tailored Ψ values (Stability–Dynamics Balance Index) for specific extreme conditions; (iii) bioinspired all-scenario adaptive materials that mimic ice-binding proteins, pressure-tolerant organisms, or self-healing biological tissues; and (iv) standardized multi-field testing protocols to enable fair comparison across different material classes and accelerate technology translation. The integration of these approaches will unlock supramolecular materials that can reliably serve in the most demanding operational scenarios, from the vacuum of deep space to the crushing pressures of the deep sea.

## Figures and Tables

**Table 1:** Classification of hydrogen bonds by binding energy.

Category	Bond Energy (kJ/mol)	Bond Energy (kcal/mol)	Typical Examples
Very strong	100–167	24–40	[F…H…F]^−^, H_2_F^+^
Strong	40–100	10–24	O–H⋯O, O–H⋯N
Moderate	20–40	5–10	C=O⋯H–O, N–H⋯O=C
Weak	<20	<5	C–H⋯O, C–H⋯π

**Table 2:** Comparative analysis of supramolecular materials with other polymer systems.

Dimension	Conventional Polymers	Supramolecular Materials	Covalent Adaptable Networks (Vitrimers)	Hybrid Systems (Supramolecular + Covalent)
Crosslink type	Covalent (permanent)	Non-covalent (reversible)	Dynamic covalent	Mixed (covalent + non-covalent)
Bond energy range (kJ/mol)	200–800	0.1–300	150–400	Wide range
Reversibility	Irreversible	Highly reversible	Moderately to highly reversible	Tunable
Self-healing	No	Room temperature/stimulus-induced	Heat/light required	Multi-mechanism
Extreme environment tolerance	High (but no repair)	Low (prone to failure)	Moderate to high	Superior (synergistic)
Recyclability	Thermoplastics: melt-processable; thermosets: not	Solvent/thermal remolding	Hot-press remoldable	Designable
Circular economy potential	Low	High	High	High

**Table 3:** Summary of imbalance mechanisms of supramolecular materials under single and multi-field coupled extreme environments.

Environment Type	Key Parameters	Primary Disruption Mechanism	Effect on Material Properties	Representative Cases with Quantitative Data	Ref.
Low temperature	≤−40 °C	Chain segment motion “freezing”; free water crystallization; exponential decrease in dynamic bond exchange rate	Loss of flexibility and self-healing; brittle fracture; loss of conductive/sensing function	Conventional polyacrylamide hydrogel completely loses elasticity at −20 °C; ionic conductive hydrogel maintains 0.49 S/m at −80 °C	[7,8,17]
Ultra low temperature	≤−80 °C	Complete freezing of molecular segments; non-covalent bond dissociation rate approaches zero; autonomous repair mode fails	Glassy or brittle solid state; damage cannot be autonomously repaired	Traditional polymers completely brittle at liquid nitrogen temperature (−196 °C); newly developed organic crystals achieve low temperature self-healing via dipole–dipole interactions	[8]
High temperature	≥150 °C	Intensified thermal motion; dissociation rate (k_off) far exceeds recombination rate (k_on); sharp decrease in “effective” crosslinking density	Sudden drop in modulus and viscosity; network topology disintegration; permanent thermo-oxidative damage	UPy quadruple hydrogen bonds significantly dissociate above 80 °C; supramolecular oligomer adhesive maintains >21 MPa shear strength at 120 °C	[9,10,22]
High salinity/corrosion	High salt (>200,000 mg/L), strong acid/base (pH < 2 or >12)	Ion shielding disrupts electrostatic interactions; water molecules compete for hydrogen bonding sites; extreme pH alters ionization state	Network swelling and disintegration; drastic deterioration of mechanical properties and function	HPAM viscosity drops sharply in high salinity brine; salt tolerant thickener maintains ≥80 mPa·s at 200,000 mg/L salinity	[10,21]
High pressure	≥10 MPa	Compression of free volume; forced alteration of chain conformation and packing; disruption of ordered assemblies driven by weak interactions	Increased rigidity; slowed kinetics; decreased response sensitivity; possible pressure induced phase transition	EHUT supramolecular polymer undergoes non entangled to entangled phase transition above 20 MPa; supramolecular gel maintains high strength under high pressure	[19,50]
Multi field coupling	Deep sea, deep earth, deep space, etc.	Positive feedback coupling of multiple mechanisms; thermo-mechano-chemical effects amplify each other; failure rate 3–10× faster than single factor exposure	Rapid material failure far exceeding superposition of individual factors; cliff like drop in performance within short time	High temperature + high salinity synergistically increases viscosity decay rate by 3–5×; deep sea (high pressure + low temperature + high salinity) poses severe comprehensive challenge	[9,51]

**Table 4:** Comprehensive summary of extreme-environment engineering applications.

Application Field	Specific Scenario	Core Material	Design Strategy	Key Performance Metrics and Quantitative Data	Innovative Breakthrough	Ref.
Deep space exploration	Spacesuit sealing layer	SPUU-DA elastomer	Orthogonal synergistic + microphase separation	Toughness 1.2 GJ·m^−3^; fracture energy 282.5 kJ·m^−2^; puncture force 136.4 N (0.7 mm film); full mechanical recovery at 60 °C/6 h	Rigid aromatic amide (DABA) + flexible adipic dihydrazide (ADH) segment synergy	[18]
	Spacesuit puncture repair	Reverlink^®^ supramolecular elastomer	Low-T_g_ dynamic network	Complete closure in 52.30 s at 0.3 bar relative pressure; leaked air volume only 0.0460 L; drying dramatically enhances self-healing	Water content control is key variable; outperforms many contemporary materials	[41]
	Ultra-wide temperature self-healing optical material	D–π–A–π–D organic crystals	Environment-adaptive dynamic bonds	Self-healing temperature range −196 °C to 150 °C (346 °C span); autonomous + non-autonomous dual-mode repair; dipole–dipole interaction driven	Crystalline intra-layer ordering + inter-layer reverse orientation create strong electrostatic attraction	[70]
	High-performance re-bondable adhesive	Solution-sheared supramolecular oligomers	Hierarchical assembly + H-bond network	Peeling work 23.6 kN/m; lap shear strength > 30.6 MPa (8× commercial structural adhesive); >21 MPa at 120 °C; multiple re-bonding cycles	Solution shearing induces multi-scale structure: ordered nanocrystals + oriented nanofibrils	[9,10,71]
Deep-sea engineering	Anti-corrosion coating	PU3/MXene composite coating	Orthogonal synergistic (rigid-flexible mismatch)	15 s underwater healing under NIR irradiation; excellent anti-corrosion performance by electrochemical impedance spectroscopy	High-density H-bond crosslinking domains + MXene photothermal conversion synergy	[72]
	Sensor encapsulation	CB[8]-based salt-enhanced coating	Environment-adaptive dynamic bonds	Binding constant increase by 1–2 orders of magnitude in high-salt environment; significantly enhanced physical barrier stability; effective metal corrosion inhibition	“Salt-enhanced” effect: high salt strengthens network	[73]
	Low-temperature Li metal battery interface layer	Ti-POM crosslinked metallo-supramolecular polymer (MSP-IPL)	Coordination crosslinked network	Electrochemical window > 5.07 V; ionic conductivity 0.485 mS·cm^−1^; Li^+^ transference number t_s_^+^ = 0.607; thickness ~1.6 μm	Ti-POM hexadentate crosslinking units bridge organic/inorganic components	[74]
	Pressure-activated sealant (CNOOC)	Differential pressure-activated supramolecular sealant	Smart adaptive network	Forms tough barrier within 150 s under 5–15 MPa differential pressure; effective sealing of thread leaks and micro-defects	Multi-phase system: hydrated colloidal particles + lamellar micro-structure; laboratory + field validation	[75]
Energy extraction	High-temperature high-salinity fracturing fluid	Hydrophobically associating polyacrylamide supramolecular gel	Orthogonal synergistic + microphase separation	Maintains viscosity > 50 mPa·s for 90 days at 150 °C in 20 × 10^4^ mg/L brine; conventional HPAM degrades within 7 days	Hydrophobic microdomains protect dynamic crosslinks from salt ions	[53]
	Salt-tolerant supramolecular thickener (STSTA)	Acrylamide copolymer + hydrophobic monomer + ionic monomer	Hydrophobic association + electrostatic interactions	Excellent solubility in 200,000 mg/L brine; ≥80 mPa·s at 0.5 wt% (170 s^−1^); shear thinning, viscoelasticity, shear recovery outperform guar gum	Dual-tail hydrophobic monomer (DHM) + non-ionic surfactant monomer (NPS) synergy	[21]
	Integrated fracturing-oil displacement supramolecular fluid	HMP–OB_14_–C_8_OH supramolecular system	Micellar-enhanced supramolecular interactions	Viscosity increase of 62 mPa·s; improved temperature, shear, and salt resistance; micellar growth promotes supramolecular network formation	Fracturing and oil displacement integration; cryo-TEM confirms micellar structure changes	[76]
	Drilling loss circulation supramolecular gel	Cellulose nanofiber/laponite-reinforced supramolecular gel	Nanofiller reinforcement + supramolecular crosslinking	Optimal formulation: 15 wt% acrylamide + 3 wt% AMPS + 5 wt% PVA + 0.30 wt% CNF + 3 wt% laponite; breakthrough pressure > 6 MPa	Maintains high strength under temperature, density, pH, drilling fluid/brine/oil invasion	[77]
Polar exploration	Polar sensor encapsulation	Zn^2+^-coordinated supramolecular elastomer	Low-T_g_ dynamic network	Self-healing efficiency ~68% at −90 °C; elongation at break 1819% at −40 °C; stable wide-temperature mechanical properties	Polybutadiene ultra-low T_g_ backbone ensures low-temperature segmental mobility	[78,79]
	Hypothermic sensing elastomer	Ion-free liquid-type supramolecular elastomer	Dual-component structural design	TCR of 8.87%/°C in −20 to −15 °C range (3–5× ordinary sensors); wireless temperature sensing accuracy comparable to infrared thermography	Ionic conductive component (anti-freeze, anti-leakage) + pendant chain damping component (signal stabilization)	[80]
	Bioinspired anti-freezing hydrogel	BMIP + IBP synergistic hydrogel	Localized ice regulation	Maintains non-frozen state at −30 °C; tensile strength 22 MPa; strain 140%; structurally intact after 40 freeze–thaw cycles	Biomimetic mechanism: BMIP promotes local nucleation + IBP inhibits ice growth; ML-enabled tactile recognition	[47]
	Ultra-low temperature ionic conductive hydrogel	Sulfobetaine + oxidized cellulose nanofibers + lithium alginate	H-bond crosslinked network	Conductivity 0.49 S/m at −80 °C; adhesion 36.73 kPa; self-healing; functionally stable > 45 days	H-bond + chemical crosslinked dual network; health monitoring and HMI in extreme cold	[48]

## Data Availability

No new data were created or analyzed in this study. Data sharing is not applicable to this article.
